# Extracellular Matrix Recycling as a Novel Plasticity Mechanism With a Potential Role in Disease

**DOI:** 10.3389/fncel.2022.854897

**Published:** 2022-03-31

**Authors:** Tal M. Dankovich, Silvio O. Rizzoli

**Affiliations:** ^1^Institute of Neuro- and Sensory Physiology, University Medical Center Göttingen, Göttingen, Germany; ^2^International Max Planck Research School for Neurosciences, Göttingen, Germany; ^3^Biostructural Imaging of Neurodegeneration (BIN) Center & Multiscale Bioimaging Excellence Center, Göttingen, Germany

**Keywords:** ECM, synapse, tenascin, recycling, neurodegeneration

## Abstract

The extracellular matrix (ECM) stabilizes neural circuits and synapses in the healthy brain, while also retaining the ability to be remodeled, to allow synapses to be plastic. A well-described mechanism for ECM remodeling is through the regulated secretion of proteolytic enzymes at the synapse, together with the synthesis of new ECM molecules. The importance of this process is evidenced by the large number of brain disorders that are associated with a dysregulation of ECM-cleaving protease activity. While most of the brain ECM molecules are indeed stable for remarkable time periods, evidence in other cell types, as cancer cells, suggests that at least a proportion of the ECM molecules may be endocytosed regularly, and could even be recycled back to the ECM. In this review, we discuss the involvement of such a mechanism in the brain, under physiological activity conditions and in relation to synapse and brain disease.

## Introduction

In the adult brain, neurons are surrounded by a lattice of extracellular matrix (ECM) molecules that coat the surfaces of neurons and fill the spaces in between synapses (Ferrer-Ferrer and Dityatev, 2018). Since ECM molecules are especially long-lived (Dörrbaum et al., 2018; Fornasiero et al., 2018), these lattices are highly robust, and are believed to stabilize neural circuits and restrict synaptic plasticity (Dansie and Ethell, 2011; Wang and Fawcett, 2012). Therefore, it is not surprising that changes to the expression and organization of various ECM molecules are associated with a plethora of psychiatric and neurodegenerative diseases (Bonneh-Barkay and Wiley, 2009; Lemarchant et al., 2013; Wen et al., 2018). The ECM could be therefore seen as a stable structure, designed to keep neuronal networks in shape, limiting their dynamics. Nonetheless, although the ECM is indeed largely restrictive to plasticity, it still retains the flexibility to be remodeled at synapses, in order to support synapse changes in the adult brain. The mechanism through which this remodeling occurs is not fully understood, but is assumed to involve a local secretion of ECM-cleaving proteases, alongside a synthesis of new ECM molecules (Dityatev et al., 2010; Krishnaswamy et al., 2019). However, while such a mechanism is likely to be sufficient for relatively infrequent events of ECM remodeling at synapses, it is arguably too metabolically expensive for synaptic changes with faster or more frequent dynamics. Recent findings propose an alternative mechanism, whereby the components of the ECM can be continually recycled at synapses (Dankovich et al., 2021). Here, we briefly review the existing paradigm for ECM remodeling and then discuss these recent findings on ECM recycling at the synapse. Since evidence also suggests that this mechanism is indispensable for synaptic function, we consider how this process may be dysregulated in brain disorders.

## The Extracellular Matrix as a Stabilizing Force in the Healthy Brain

During the final stages of brain development, the brain ECM undergoes a profound change in its molecular and spatial composition. A central aspect of this change is the appearance of dense ECM coats named “perineuronal nets” (PNNs) around the somas and proximal dendrites of a subset of neurons, in particular around parvalbumin-expressing inhibitory neurons (Härtig et al., 1992; Ruoslahti, 1996; Yamada and Jinno, 2013). These coats are composed of hyaluronic acid chains that exude from the surface of the neurons and, in turn, bind a family of secreted proteoglycans called lecticans (that includes neurocan, brevican, versican, and aggrecan). These lecticans are cross-linked by their binding partner tenascin-R, and their association with hyaluronan is further stabilized through multiple interactions with hyaluronan and proteoglycan link proteins (HAPLNs) (Ruoslahti, 1996; Sorg et al., 2016). The appearance of PNNs coincides with the closure of the critical period of plasticity, when neuronal circuits are highly sensitive to experience (Fawcett et al., 2019). As a result, it is widely believed that PNNs regulate the switch from juvenile to adult plasticity by restricting the reorganization of neural circuits (Gundelfinger et al., 2010). In line with this notion, destroying PNNs by injecting ECM-cleaving enzymes into specific brain regions can revive juvenile forms of plasticity in rodents. One such example is the rejuvenation of ocular dominance plasticity, where deprivation of visual input into one eye leads to a weakening of the neural responses it evokes, alongside an increase in the responses evoked by the non-deprived eye (Pizzorusso, 2002). Similar treatments have also rendered drug addiction and fear memories susceptible to erasure by extinction, a quality that is unique to juvenile animals (Gogolla et al., 2009; Xue et al., 2014).

The stabilizing effect conferred by the PNNs should be essential to normal brain function, since the role of PNNs in stabilizing neural circuits contributes critically to the maintenance of a balance between neuronal excitation and inhibition. In addition, the nets probably also act as a physical and chemical barrier that protects the neurons from oxidative stress and other toxic molecules (Miyata et al., 2007; Suttkus et al., 2012, 2016). Expectedly, alterations in the structure of PNNs have been observed in a number of CNS diseases, several examples being schizophrenia, epilepsy, Alzheimer’s disease and amyotrophic lateral sclerosis (ALS) (Bonneh-Barkay and Wiley, 2009; Lemarchant et al., 2013; Bitanihirwe and Woo, 2014; Wen et al., 2018; Kaushik et al., 2021).

Besides the dense PNN formations, advances in imaging resolution have revealed that the ECM is ubiquitous at the neuronal surface, albeit in a looser configuration, and that ECM molecules can also be found in tight proximity to synapses (Dityatev et al., 2006). As with PNNs, this “loose” perisynaptic ECM appears to be equally important for maintaining the stability of mature synapses. For example, enzymatic cleavage of hyaluronan, the structural backbone of neural ECM lattices (Ruoslahti, 1996), was shown to increase the lateral mobility of synaptic AMPA-type glutamate receptors (Frischknecht et al., 2009), which suggests that the appearance of ECM at mature synapses assists in retaining neurotransmitter receptors in the synaptic membrane. In addition, enzymatic cleavage of lecticans was shown to enhance the motility of dendritic spines, as well as the outgrowth of dendritic spine heads, suggesting that the ECM is also restrictive to structural changes to the synapse (Orlando et al., 2012; de Vivo et al., 2013). Besides acting as a physical barrier, evidence also suggests that ECM molecules can interact with the synaptic transmission machinery and potentially promote the organization of these proteins at the synapse. This includes direct interactions with ion channels and neurotransmitter receptors, as well as indirect interactions through synaptic ECM receptors such as integrins (Dityatev and Schachner, 2003). It is therefore to be expected that a deficiency in various ECM molecules or their receptors manifests in dysfunctional synaptic transmission and plasticity (e.g., Qiu et al., 2006; Bukalo et al., 2007; Roszkowska et al., 2016).

## Remodeling of the Extracellular Matrix Permits Synaptic Plasticity

**FIGURE 1:**
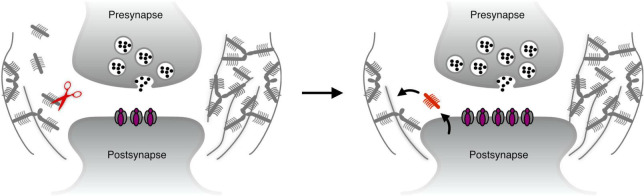
The dominant paradigm for ECM remodeling – proteolytic cleavage. Left: when synaptic plasticity is initiated, ECM cleaving proteases such as MMP9 (Michaluk et al., 2011) may be translated and/or secreted locally at the synapse. Right: the synapse undergoes structural plasticity, and new molecules are secreted to re-stabilize the perisynaptic ECM.

The discussions in the previous section suggest that the ECM predominantly restricts synaptic plasticity. However, since synapses undergo structural changes long after maturity (Yang et al., 2019), the ECM at synapses needs to be susceptible to transient remodeling during such events. The dominant paradigm for ECM remodeling during synaptic plasticity is through proteolytic cleavage, and this process is described roughly as follows: a surge in synaptic activity results in localized activation of ECM-cleaving enzymes (e.g., through translation from mRNA that is locally present at the synapse). The resulting cleavage permits the synapse to undergo structural changes (e.g., the growth of the postsynaptic head), and may also expose latent sequences in the ECM molecules that activate synaptic receptors to further boost plasticity-related changes. Finally, proteolytic activity is inhibited, and *de novo* synthesized ECM molecules are secreted to replace the previously cleaved molecules, thus allowing the structural changes to the synapse to persist (Figure 1).

The best-studied example of proteolysis-mediated plasticity involves the activity of the ECM-cleaving enzyme matrix metalloproteinase 9 (MMP9). The activity-dependent increase in MMP9 expression was shown to be necessary for long-term potentiation (LTP) maintenance in the CA1 region of the hippocampus (Nagy et al., 2006; Wang et al., 2008). Upon increased neuronal activity, MMP9 mRNA is translated and secreted locally at synapses (Zagulska-Szymczak et al., 2001; Gawlak et al., 2009; Dziembowska et al., 2012). Once activated, MMP9 cleaves a variety of targets in the synaptic extracellular space, some examples being the synaptic adhesion molecules neuroligin-1 and intercellular adhesion molecule-5 (ICAM-5) (Peixoto et al., 2012; Kelly et al., 2014), as well as the ECM molecule aggrecan (Mercuri et al., 2000). One effect of MMP9 activity at synapses is an enlargement of the dendritic spine head, which is a structural hallmark of LTP (Yuste and Bonhoeffer, 2001). Besides cleaving synaptic ECM to presumably facilitate spine enlargement, MMP9 activity also results in the activation of postsynaptic β1 integrin receptors, whose signaling further promotes remodeling of the actin cytoskeleton (Wang et al., 2008; Michaluk et al., 2011). Finally, once MMP9 has completed its role, it is presumably inhibited by TIMP1, a member of the tissue inhibitors of metalloproteinases (TIMP) family (Okulski et al., 2007; Magnowska et al., 2016). Lastly, the cessation of plasticity is expected to be accompanied by the secretion of newly synthesized ECM molecules. Indeed, studies have demonstrated that the expression of various ECM molecules is transiently upregulated by increased neuronal activity (Heck et al., 2004; Niekisch et al., 2019; Rao-Ruiz et al., 2019).

Though comparatively less studied, additional enzymes with roles in synaptic plasticity are also emerging. For example, two members of the MMP family, MMP3 and MMP7, have both been shown to drive activity-dependent changes in synaptic structure (Bilousova et al., 2006; Wójtowicz and Mozrzymas, 2014; Aerts et al., 2015; Brzdąk et al., 2017). Recently, the protease cathepsin-S was suggested to remodel PNNs in a circadian manner, which would presumably allow synapses to be modified during sleep (Pantazopoulos et al., 2020; Delorme et al., 2021; Harkness et al., 2021). Additional proteases, including neurotrypsin and members of the “a disintegrin and metalloproteinase with TSP motifs” (ADAMTS) family, may also be involved in mediating synaptic plasticity. Since this topic is beyond our scope, we refer the reader to excellent reviews on the involvement of ECM-cleaving enzymes in plasticity (Dityatev et al., 2010; Gottschall and Howell, 2015; Beroun et al., 2019).

## Can Proteolysis Support the Physiological Frequency of Extracellular Matrix Remodeling at Synapses?

If structural changes to synapses were rare events, it would be reasonable to assume that synaptic ECM is destroyed and resynthesized with every change. However, a growing number of studies are revealing that synapse size and morphology are constantly fluctuating, on a timescale of minutes to hours (e.g., Berning et al., 2012; Willig et al., 2014; Wegner et al., 2018). Such studies have taken advantage of super-resolution tools to investigate the dynamics of synapses in live brain tissue, and have shown frequent changes in synaptic position and morphology that had remained hidden for previous studies performed by conventional microscopy tools with resolutions substantially above the synapse size. This correction in our perception of synapse changes *in vivo* implies that one now needs to wonder about how the ECM can cope with the normal physiology of the synapses every day, and not just with rare plasticity events. The notion that the ECM is turned over (by enzymatic degradation and new synthesis) at an equally rapid rate to the synapse changes is not consistent with the fact that its components are some of the longest-lived molecules in the brain (ECM components have, on average, a half-life of over 1 month in rodents *in vivo*) (Toyama et al., 2014; Dörrbaum et al., 2018; Fornasiero et al., 2018; Heo et al., 2018). Hence, while proteolysis may accompany infrequent events of structural synaptic plasticity, an additional remodeling mechanism is needed to account for the more frequent changes to synapses that take place even in the absence of plasticity-triggering stimuli. A compelling possibility, discussed in the next section, is that the ECM at synapses can be remodeled through the recycling of its components, without the need for proteolysis and *de novo* synthesis.

## Recycling as an Alternative Mechanism for Extracellular Matrix Remodeling

**FIGURE 2:**
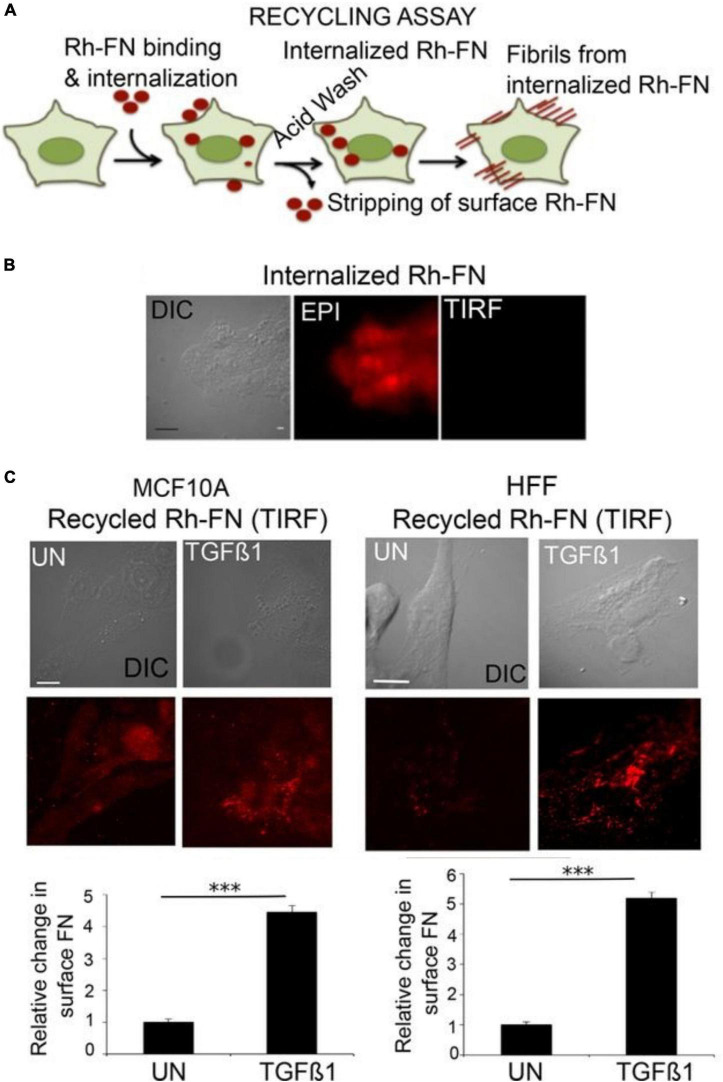
Fibronectin recycling in epithelial cells. **(A)** Schematic of the assay used to assess fibronectin recycling. Live cells were incubated with Rh-FN (rhodamine-labeled fibronectin) for 30 min at 37°C. The cells were then stripped by acid washing to remove surface-bound molecules, so that only signal from internalized Rh-FN remains. Afterward, the cells were incubated in medium containing TGF-β1 for 1 h at 37°C, and then imaged using total internal reflection (TIRF) microscopy to assess the appearance of the internalized Rh-FN at the cell surface. **(B)** Following the incubation with Rh-FN, the cells were imaged both in TIRF mode or with epifluorescence to verify that the molecules had internalized. **(C)** The amount of resurfacing Rh-FN was assessed in TGF-β1-treated MCF10A breast epithelial cells or human foreskin fibroblasts (HFF), demonstrating that TGF-β1 induces significant recycling of FN in both cell types. Scale bar = 5 μm. The quantifications below the images were performed using the ImageJ 3D Object Counter plug-in. *N* = 10 cells per condition from at least three independent experiments. ***p < 0.001. Adapted from Varadaraj et al. (2017) with permission from the American Society for Cell Biology.

The concept of ECM re-internalization from the extracellular space is not entirely unprecedented. In non-neural cells, multiple ECM molecules have been shown to undergo endocytosis after binding cell-surface receptors such as integrin, dystroglycan and CD44 (Coopman et al., 1996; Tammi et al., 2001; Shi and Sottile, 2008; Lobert et al., 2010; Leonoudakis et al., 2014). The common belief among these studies was that these internalized molecules were targeted for degradation, and so they typically did not examine whether a subset of these molecules might eventually be secreted once again to the extracellular space. A number of years ago, a study by Varadaraj and colleagues demonstrated a complete recycling loop for the ECM molecule fibronectin in fibroblasts and in epithelial cells. Using an acidic buffer treatment to strip away cell-surface molecules (thus enabling the investigators to discern the intracellular population), the authors showed that stimulation with TGF-beta induces uptake of fluorescently labeled fibronectin. Subsequently, these molecules were recycled back to the surface, where they were successfully incorporated into fibrils (Figure 2). The internalization of fibronectin was found to be dependent on both α5β1 integrin receptors and the type II TGF-β receptor (Varadaraj et al., 2017). To our knowledge, this is the first establishment of a link between cellular dynamics (i.e., fibrillogenesis, a process involved in migration and proliferation: Schwarzbauer and DeSimone, 2011) and ECM recycling.

**FIGURE 3:**
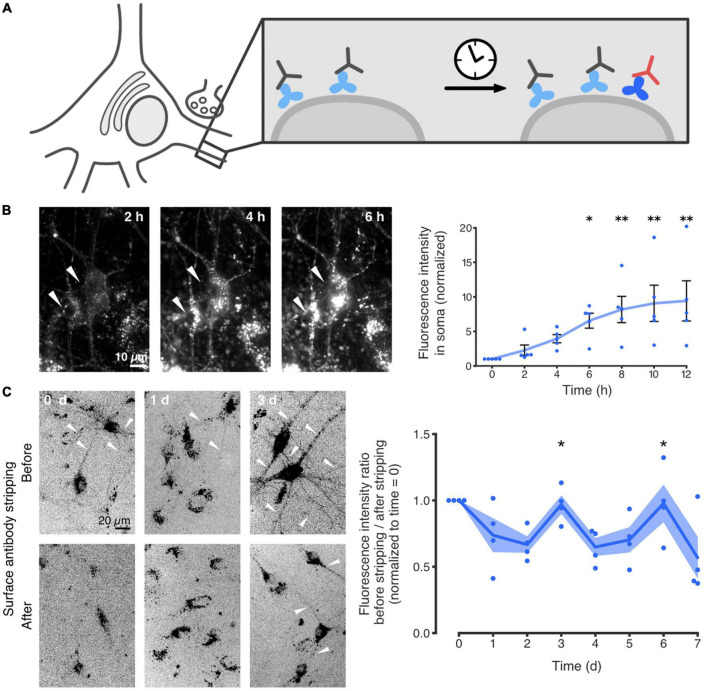
TNR internalization and recycling in hippocampal neurons. **(A)** Schematic of the experimental assay for the labeling of recycling molecules. Live cultured neurons were incubated with non-fluorescent antibodies (gray) to block all of the surface TNR epitopes (light blue). After a period of time (4–6 h), the neurons were incubated with fluorophore-conjugated TNR antibodies (red) to label any “newly emerged” TNR epitopes which were not present at the neuronal surface beforehand (dark blue). Since the half-life of TNR in these cultures is ∼7 days (Dörrbaum et al., 2018), the amount of newly synthesized TNR molecules that emerge at the surface should not be significant. The fluorescently labeled TNRs can be subsequently followed in imaging experiments. **(B)** Time-lapse imaging of newly emerged TNR epitopes over 12 h. It is evident that the TNR epitopes are accumulating in the neuronal somas (two examples are indicated by the white arrowheads), demonstrating a significant internalization of these molecules. Scale bar = 10 μm. The plot shows a quantification of the mean TNR fluorescence intensity in multiple neuronal somas, normalized to the intensity at *t* = 0 h. A visible increase over 12 h is observed, confirming the observation that the molecules are internalized. *N* = 5 independent experiments, with 1–4 neurons each. Statistical significance was evaluated using the Friedman test (χ^2^_6_ = 25.46, ***p < 0.001), followed by Dunn’s multiple comparisons test (*p = 0.033, **p = 0.005, **p = 0.005 and **p = 0.002 for the 6, 8, 10, and 12-h timepoints, respectively). **(C)** The proportion of newly emerged TNR epitopes at the neuronal surface was measured over 6 days, by imaging before and after a treatment with proteinase K to strip away cell-surface molecules. Immediately after labeling (“0 days”), virtually no neurites were visible after stripping, indicating that the majority of the newly emerged TNR molecules are at the surface. On day 1, the stripping had little effect, indicating that many TNR molecules had internalized. On day 3, neurites were once again visible before but not after stripping, indicating that a large amount of TNR molecules had returned to the neuronal surface. Scale bar = 20 μm. The plot shows a quantification of the fluorescence ratio before/after stripping, normalized to *t* = 0 days. The peaks at days 3 and 6 indicate that TNR recycles with a periodicity of ∼3 days. *N* = 4 independent experiments. Statistical significance was evaluated with the Kruskal-Wallis test (days 2–4: *H*_2_ = 8.29, *p = 0.016, days 4 – 6: *H*_2_ = 6.74, *p = 0.036), followed by Fisher’s LSD (“3d” vs. “2d”: *p = 0.046; “3d” vs. “4d”: **p = 0.005; “6d” vs. “5d”: *p = 0.022; “6d” vs. “7d”: *p = 0.028). All data represent the mean (lines) ± SEM (panel **B**: whiskers; panel **C**: shaded regions), with dots indicating individual experiments. Adapted from Dankovich et al. (2021) with permission from Springer Nature (http://creativecommons.org/licenses/by/4.0/).

Recently, we reported that the neuronal ECM is similarly capable of being recycled. More specifically, we showed that the ECM glycoprotein tenascin-R (TNR) is internalized from the synaptic extracellular space, and eventually resurfaces at synapses. The study largely relied on an immunostaining-based assay for labeling TNR molecules that recently emerged at the surface of live neurons. Using this approach, it was demonstrated that these dynamic molecules are endocytosed by neurons, undergo retrograde trafficking to the soma, and eventually reappear at the neuronal surface (Figure 3). Further experiments relying on super-resolution imaging demonstrated that this process occurs preferentially at synapses (Dankovich et al., 2021).

Our observation that TNR recycling lasts ∼3 days is particularly surprising, considering that most surface molecules are known to recycle within minutes to hours (e.g., Bretscher, 1989; Koenig and Edwardson, 1997; Bridgewater et al., 2012). Further immunostaining experiments revealed that the internalized TNRs colocalize with markers for both the Golgi apparatus and endoplasmic reticulum in the neuronal somas. In addition, metabolic labeling of glycoproteins demonstrated that the recycling TNR molecules appear to incorporate new glycans (i.e., become re-glycosylated) following their trafficking through these organelles. Taken together, these findings may provide a partial explanation for this exceptionally long recycling loop.

**FIGURE 4:**
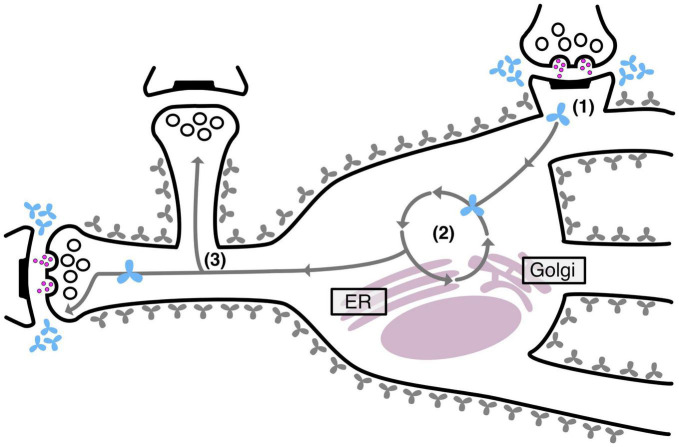
TNR recycling mechanism and function in neurons. Neurons contain two pools of TNR molecules: a stable pool (gray molecules) and a recycling pool, which is enriched at synapses (blue molecules). After their internalization at synapses (1), the recycling TNR molecules are trafficked to the Golgi apparatus and the endoplasmic reticulum, where they appear to undergo a re-glycosylation (2). At the end of their route, these molecules are once again trafficked to synapses (3). Stronger synapses (with a larger pool of actively recycling presynaptic vesicles or with larger postsynaptic spine heads) have more recycling TNR molecules in their vicinity.

Interestingly, we also observed that neurons effectively maintain two separate pools of TNR molecules: a stable pool that remains embedded in the ECM and a recycling pool that shuttles back and forth between the neuronal intra- and extracellular space. As mentioned, this recycling pool was found to be enriched at synapses, but in addition, it was also found to be significantly more abundant at highly active synapses. This latter finding strongly supports the notion that ECM recycling constitutes a mechanism for ECM remodeling during synaptic plasticity. Importantly, it was found that disrupting this recycling process also seriously modified synaptic structure and transmission (Dankovich et al., 2021). The findings of this study are summarized in Figure 4, below.

In addition to the metabolic benefits of ECM recycling, which frees the cells from the need to repeatedly synthesize both the ECM molecules and the ECM-cleaving enzymes, this mechanism could also serve an additional function, besides remodeling the extracellular space around synapses. As mentioned above, ECM remodeling through proteolysis may reveal latent sequences on ECM molecules that activate synaptic receptors and trigger plasticity-related intracellular signaling cascades (Dityatev et al., 2010). In a parallel fashion, it is possible to imagine that the internalization of *intact* ECM molecules by neurons is necessary for the activation of intracellular signaling cascades, perhaps through the interaction of these ECM molecules with co-trafficked proteins. Furthermore, if it is revealed that recycling ECM molecules are secreted and internalized by different neurons (e.g., from the pre- to the post synapse), this mechanism may constitute a novel form of *trans-*neuronal communication, an interesting possibility that may be explored in further studies.

## Potential Implications for Brain Diseases

Since changes to the expression of ECM molecules are known to accompany a wide variety of brain diseases, it would be expected that perturbations to ECM recycling similarly manifest themselves in disease. In agreement with this notion, the proportion of somatic intracellular TNR molecules was increased in a mouse model of epilepsy (Dankovich et al., 2021). This accumulation of intracellular TNR is unlikely to be solely the result of neuronal damage, since this effect was not observed in a model of familial Alzheimer’s disease, where neuronal damage is also prominent (Oakley et al., 2006). Interestingly, seizures have also been shown to upregulate the expression of a number of ECM molecules, including TNR (Ulbrich et al., 2021). While it is possible that these outcomes are causally linked, future studies should investigate this in greater detail and attempt to pinpoint dysregulated processes that modulate TNR recycling (as well as other ECM molecules). Other disease models that involve known modifications of mature ECM and thus warrant further investigation include multiple sclerosis, Alzheimer’s disease, ALS, Parkinson’s disease, schizophrenia and bipolar disorder (Bonneh-Barkay and Wiley, 2009; Lemarchant et al., 2013; Pantazopoulos and Berretta, 2016; Wen et al., 2018).

Besides its involvement in maintaining mature neural circuits, the ECM also plays an important developmental role, including the support of neurite extension, neuronal migration and cortical folding (Long and Huttner, 2019; Amin and Borrell, 2020). As for mature ECM, it is probable that some of these early roles may be supported by a putative recycling of ECM molecules expressed in the developing brain. For example, dysregulations of the ECM protein reelin, which plays an important role in neuronal migration, have been linked to various disorders such as autism spectrum disorder (ASD), schizophrenia and bipolar disorder (Ishii and Maeda, 2008; Lakatosova and Ostatnikova, 2012). The observation that reelin molecules can be internalized through an interaction with their receptors VLDLR and ApoER2 hints to the possibility that a portion of these molecules can be recycled back to the membrane. The ECM protein laminin is similarly known to be dysregulated in neuronal migration disorders, and was also shown to be internalized in non-neuronal cells (Barak et al., 2011; Radmanesh et al., 2013; Radner et al., 2013; Leonoudakis et al., 2014). The prospect of ECM recycling in early development should be investigated in greater depth in the future, including the possible involvement of such perturbations in various neurodevelopmental disorders.

## Conclusion and Future Directions

Synaptic plasticity in the adult brain is believed to be accompanied by a remodeling of the local ECM, presumably through proteolysis and *de novo* synthesis of ECM molecules (Dityatev et al., 2010; Krishnaswamy et al., 2019). However, while this form of remodeling may account for infrequent instances of plasticity, it is likely to be too metabolically costly to support regular fluctuations to synaptic structure, as have been demonstrated to occur by numerous studies (Berning et al., 2012; Willig et al., 2014; Wegner et al., 2018). Importantly, such a mechanism would not be in line with the long lifetimes of ECM molecules (Toyama et al., 2014; Dörrbaum et al., 2018; Fornasiero et al., 2018; Heo et al., 2018). A possible solution to this problem arose from a recent study describing a novel mechanism of ECM remodeling at synapses through the recycling of the ECM molecule TNR (Dankovich et al., 2021). It was shown that a targeted disruption of this mechanism severely modified synaptic function, suggesting that dysregulated recycling *in vivo* is highly likely to play a role in disease.

An interesting line of future research would be to assess potential dysfunctions that are a direct outcome of perturbations to ECM recycling. This could be assessed by treating animals with large aggregates of antibodies directed against ECM molecules, as we performed in our study *in vitro* (Dankovich et al., 2021). Since it was found that interrupting TNR recycling modified evoked synaptic transmission as well as dendritic spine head size, it is likely that potential dysfunctions would involve similar phenotypes. It is possible to imagine, for example, that disrupting ECM recycling would reduce neuronal excitability. In principle, if certain diseases are found to have augmented recycling, inhibiting this process may have potential therapeutic benefits, albeit one would need to develop more suitable inhibitors than antibody aggregates, which would probably have major difficulties in penetrating into the brain, and would probably also have severe inflammation-inducing effects. For the reverse situation, it would be beneficial to develop techniques to enhance ECM recycling, for example, by interfering with the interaction of these molecules with extracellular binding partners. Such a treatment may act to reduce neuronal excitability and could therefore have therapeutic potential in disorders such as epilepsy. Once sufficient tools are developed for probing ECM recycling in animals *in vivo*, such investigations will be an exciting possibility for future studies.

In conclusion, while further experiments are needed to establish whether ECM recycling is a widespread mechanism among multiple molecules, the discovery of a novel constitutive process in neurons opens up an exciting new avenue of research in models of brain disease. We expect that future studies investigate the involvement of ECM recycling in disease in greater detail, as well as potential therapeutic treatments that target this process.

## Author Contributions

TD and SR conceived the manuscript. TD wrote the manuscript. SR revised the manuscript. Both authors contributed to the article and approved the submitted version.

## Conflict of Interest

The authors declare that the research was conducted in the absence of any commercial or financial relationships that could be construed as a potential conflict of interest.

## Publisher’s Note

All claims expressed in this article are solely those of the authors and do not necessarily represent those of their affiliated organizations, or those of the publisher, the editors and the reviewers. Any product that may be evaluated in this article, or claim that may be made by its manufacturer, is not guaranteed or endorsed by the publisher.
